# Impact of Dietary Patterns on the Lipidemic Profile and the Cardiovascular Risk in Stage 1 Hypertension: A Post Hoc Analysis of the HINTreat Trial

**DOI:** 10.3390/nu17162632

**Published:** 2025-08-14

**Authors:** Anastasios Vamvakis, Antonios Lazaridis, Maria G. Grammatikopoulou, Anastasia Malliora, Kyriaki Tsiroukidou, Christos Tzimos, Andrea Di Blasio, Pascal Izzicupo, Eugenia Gkaliagkousi

**Affiliations:** 1 Department of Nutritional Sciences & Dietetics, Faculty of Health Sciences, Hellenic Mediterranean University of Crete, Tripitos Area (2nd km Sitia–Palekastro), GR-72300 Sitia, Greece; 2 1st Department of Internal Medicine, Papageorgiou General Hospital of Thessaloniki, Faculty of Health Sciences, School of Medicine, Aristotle University of Thessaloniki, GR-56403 Thessaloniki, Greece; spanbiol@hotmail.com; 3 Immunonutrition Unit, Department of Rheumatology and Clinical Immunology, Faculty of Medicine, School of Health Sciences, University of Thessaly, Biopolis, GR-41110 Larissa, Greece; mgrammat@uth.gr; 4 3rd Department of Internal Medicine, Papageorgiou Hospital, Faculty of Health Sciences, School of Medicine, Aristotle University of Thessaloniki, Ring Road, Nea Efkarpia, GR-56403 Thessaloniki, Greece; anamalliora@yahoo.gr (A.M.); egkaliagkousi@auth.gr (E.G.); 5 3rd Department of Pediatrics, Hippokration General Hospital of Thessaloniki, Faculty of Health Sciences, School of Medicine, Aristotle University of Thessaloniki, GR-54642 Thessaloniki, Greece; ktsiroukidou@gmail.com; 6 Northern Greece Statistics Directorate, Hellenic Statistical Authority, 218 Delfon Str, GR-54646 Thessaloniki, Greece; ctzimos@gmail.com; 7 Department of Medicine and Aging Sciences, University “G. D’Annunzio” of Chieti-Pescara, Via dei Vestini 31, 66100 Chieti, Italy; andiblasio@gmail.com (A.D.B.); pascal.izzicupo@unich.it (P.I.)

**Keywords:** diet, dietitian, nutrition, hypercholesterolemia, Mediterranean Diet, DASH diet, dietary inflammatory index, lifestyle intervention, medical nutrition therapy

## Abstract

**Background/Objectives**: In hypertension (HTN), lifestyle modification is important for controlling blood pressure (BP) and lipidemic profile. The HINTreat trial showed that an anti-inflammatory diet was associated with improved endothelial function, after six months of intensive nutritional treatment. **Methods**: This post hoc analysis of the HINTreat trial examined how adherence to various nutritional patterns like the Mediterranean Diet (MedDiet), the Dietary Approaches to Stop Hypertension (DASH) diet, and anti-inflammatory diet, had impact on the blood lipids profile and the CVD risk. Patients with stage 1 HTN, allocated either on intensive lifestyle treatment (ILT) or usual care (UC) standard treatment, participated in the analysis. From the original sample size of the HINTreat trial, all patients that were prescribed lipid lowering medication at any time of the study period were excluded from the total analysis; thus, the intervention and the control groups consisted of 33 and 28 patients, respectively. Nutritional intakes were assessed with repeated 24 h recalls from the previous day, and dietary indexes and scores were calculated as follows: MedDiet score, DASH index, and Dietary Inflammatory Index (DII). After six months of intervention, changes in the nutritional indexes and their effect on the lipidemic profile and CVD risk were analyzed. **Results**: In the ILT group, reductions were noted in Ambulatory Blood Pressure Monitoring (ABPM) for day systolic BP (SBP) (−12.7 mmHg) and diastolic BP (DBP) (−8.4 mmHg), total cholesterol (TC) (−35.4 mg/dL), triglycerides (TG) (−21.4 mg/dL), LDL cholesterol (LDL-C) (−27.5 mg/dL) concentrations, and CVD risk score (−1.5%), *p* < 0.001 for all. Multiple regression analysis showed that dietary quality indices independently influenced improvements in blood lipid profile and cardiovascular disease (CVD) risk among patients receiving ILT. Specifically, a higher Mediterranean Diet (MedDiet) score was significantly associated with reductions in TC (B = −7.238, *p* < 0.001), TG (B = −4.103, *p* = 0.035), and LDL-C (B = −6.431, *p* = 0.004). The DASH index was positively associated with TG levels (B = 9.913, *p* = 0.010), suggesting a more complex relationship that may require further investigation. In addition, DII was positively associated with increased CVD risk (B = 0.973, *p* < 0.001). **Conclusions**: The findings suggest that ILT can improve BP levels, target blood lipids concentrations, and reduce CVD risk in patients with stage 1 HTN.

## 1. Introduction

Essential hypertension (EH) is one of the leading preventable and modifiable causes of atherosclerotic cardiovascular disease (CVD), contributing to global mortality [1,2]. Hypertension (HTN) affects about one third of the population and has doubled over the last 30 years [3,4]. In the year 2023, the World Health Organization (WHO) reported that of 54% of adults who were diagnosed with HTN, a “silent killer”, 42% received treatment and only 21% had their blood pressure (BP) controlled [5].

Elevated BP, among other modifiable parameters, like smoking, diabetes mellitus (DM), obesity and dyslipidemia, is strongly associated with cardiovascular outcomes, increasing the risk for coronary artery disease (CAD), stroke, heart failure, atrial fibrillation, and peripheral vascular disease [6]. Meta-analysis showed that a reduction of 10 mmHg in elevated systolic BP (SBP) is associated with a 15–20% reduction in the risk of CAD and a 25–30% reduction in the risk of stroke [7]. Subclinical atherosclerosis, that is influenced both by non-modifiable (age, sex, genetic heritage) and modifiable factors (high BP, DM, dyslipidemia, obesity, physical inactivity, and smoking) [8], can also lead to vascular changes inducing arterial stiffness and endothelial dysfunction, predecessors of CVD [9].

Early detection and treatment of dyslipidemia are crucial for preventing atherosclerosis in people with HTN, who often exhibit triglycerides (TG) and low-density lipoprotein cholesterol (LDL-C) [10]. Increased LDL-C and TG, along with lower levels of high-density lipoprotein (HDL) are considered strong risk factors for CVD, while additionally, age and sex can also affect the lipidemic profile of patients with HTN [11]. Alteration in the lipoprotein profile is often found in patients with HTN and elevated levels of TG and LDL-C are observed during all grades of HTN, with a positive association between serum lipid levels and HTN prevalence [12]. Therefore, early recognition of dyslipidemia is of major concern [13] and assessment of the total CVD risk is important to intensify the relative treatment [14,15]. Monitoring HTN-mediated target organ damage like vascular, renal, and endothelial function, screening biochemical and lipidemic profile is essential for the prevention of CVDs [16].

Non-pharmacological treatment of elevated BP and abnormal lipid profiles, including dietary interventions and increased physical activity (PA), is fundamental in controlling both HTN and dyslipidemia [14,15]. Evidence suggests that controlling the diet, obtaining normal body weight, and participating systematically in PA are important modifications for the prevention of atherosclerosis and a dose dependent association between lifestyle modification and lipidemic profile has been shown [17,18]. In addition, numerous nutritional patterns, such as the Mediterranean Diet (MedDiet), the Dietary Approaches to Stop Hypertension (DASH), or anti-inflammatory/antioxidant diets have been associated with improvements in dyslipidemia and cardiometabolic risk parameters [19,20,21,22]. A healthy diet is a fundamental component in the prevention of atherosclerotic CVD (ASCVD). Scientific evidence supports that dietary patterns rich in fruits, vegetables, legumes, nuts, seeds, plant-based proteins, and fatty fish, and low in saturated fats, dietary cholesterol, salt, refined grains, and ultra-processed foods, are beneficial [23].

Choosing the appropriate dietary pattern to target specific aspects of the blood lipid profile may be an effective strategy [24], as not all dietary approaches exert the same cumulative effect on individual lipid markers [25]. Research suggests that different dietary patterns are associated with varying impacts on blood lipid levels, and that a dose–response relationship exists between the degree of adherence to these diets and the improvement in the lipidemic profile [26,27]. Recommended dietary models for reducing CVD risk in individuals with HTN, such as the MedDiet, the DASH diet, and anti-inflammatory or antioxidant-rich diets emphasize the consumption of fruits, vegetables, and minimally processed foods that are low in salt and dietary cholesterol. However, these patterns differ in their content of micronutrients and phytochemicals, which may influence their effectiveness in managing hyperlipidemia and reducing CVD risk [28,29]. So, choosing the most effective nutritional pattern in relation to treatment target might be a more specific beneficial strategy. To our knowledge, there are no clinical trials evaluating those three nutritional indexes simultaneously for the effect on the lipidemic profile of patients newly diagnosed with stage 1 HTN.

## 2. Materials and Methods

### 2.1. Ethical Clearance and Protocol Registry

This post hoc analysis conducted using data from the HINTreat randomized controlled trial (RCT). HINTreat RCT was a prospective, randomized, single-blind, parallel clinical study of patients—controls that took place in Northern Greece and specifically in Thessaloniki. The study was conducted based on the guidelines and principles of the Declaration of Helsinki. The protocol was registered with approval number (IRCT20200307046715N1, https://trialsearch.who.int/ (accessed on 14 March 2020)) and was approved by the Ethics Committee of the Aristotle University of Thessaloniki with approval number (2.169/23 March 2016). The study was conducted at the Laboratory of Small and Large Vessel Hypertension, Third University Department of Internal Medicine, Aristotle University of Thessaloniki, Papageorgiou General Hospital. Prior to the initiation of the study, all subjects were informed and signed a consent form to participate. Information about the original study protocol and participants can be found elsewhere [30].

### 2.2. Participants and Interventions

The present post hoc analysis aims to evaluate the effect of adherence of different dietary patterns on changes in serum blood lipids and CVD risk following a six-month intensive lifestyle treatment (ILT) intervention in stage 1 HTN.

A total of 91 adult men and women participated in the study. Participants were newly diagnosed with stage 1 HTN and were enrolled into one of two groups: the ILT intervention group (n = 38), which received intensified nutrition and PA counseling, or the usual care (UC) control group (n = 38), which received standard treatment in accordance with the ESH guidelines [31].

For the scope of this post hoc analysis, 5 patients from the ILT group and 10 patients from the UC group were excluded from the statistical analysis, due to antihyperlipidemic medication prescribed during the study period. Thus, the ILT group and the UC group consisted of 33 and 28 patients, respectively. Baseline characteristics of the participants in this post hoc analysis, for each group, are presented in Table 1.

### 2.3. BP Measurements

BP measurements included office BP and ambulatory BP monitoring (ABPM), using a validated oscillometric device (Microlife Exact BP, Microlife AG, Widnau, Switzerland) and a Mobil-O-Graph Holter device (I.E.M. GmbH, Stolberg, Germany), for which at least 70% of the readings were successful [32,33]. EH was diagnosed when office BP > 140/90 mmHg, and when brachial ABPM > 135/85 mmHg, on average daytime, according to the time ESH guidelines [31]. For this post hoc analysis, the updated ESH criteria for the classification were used [15].

### 2.4. Lipidemic Profile

Morning fasting blood samples were collected to assess biochemical and serum blood lipids analysis, including TC, TG, HDL, and LDL-C.

### 2.5. CVD Risk Stratification

The Hellenic Risk Score (HRS) II was used to estimate fatal ASCVD based on sex, age, smoking status, SBP, and TC. According to their HRS and baseline CVD history, participants were then stratified into four risk categories as follows: 1: Low Risk (LR) = <1%, 2: Moderate Risk (MR) = ≥1% and <5%, 3: High Risk (HR) = ≥5% and <10%, 4: Very High Risk (VHR) = ≥10% [34]. The presence of dyslipidemia was diagnosed according to the guidelines of the Hellenic Atherosclerosis Society [35].

### 2.6. Dietary Assesment and Calculation of Nutritional Indexes

Dietary data were obtained from repeated 24 h recalls, which were assessed by a registered dietician–nutritionist who interviewed the patients for each recall. Nutritional indexes scores were computed from the 24 h repeated recalls analysis for the MedDiet score [36], the DASH index [37], and the DII [38]. All scores were used to estimate the adherence to the related dietary pattern, as followed by the patients included in this post hoc analysis. Inter-rater reliability for the 24 h dietary recall data was not examined in the context of this study.

#### 2.6.1. MedDiet Score

The MedDiet score is an indicator that describes the MedDiet pattern and captures the degree of adherence. Its design was based on the Mediterranean food pyramid and its total score ranges from 0 to 55 for the total of 11 food categories (non-refined cereals, potato, fruits, vegetables, legumes, fish, meat, poultry, cheese, olive oil in cooking, alcohol). A score from 0 to 5 is given to each category depending on the frequency of consumption per day or week and in relation to whether a food characterizes the MedDiet (positive correlation of score with consumption) or is not often found in the MedDiet (negative correlation of score with consumption). For wine intake, consumption of 1–2 glasses per day is scored with 5, while either zero consumption or consumption of more than seven glasses of wine per day is scored with 0. A higher score is associated with better adherence to the MedDiet [36].

#### 2.6.2. DASH Index

The DASH index assesses the adherence to the DASH diet, and its calculation is based on consumption of 8 key components: fruits, vegetables, nuts and legumes, low fat dairy, whole grains, sodium, sweetened beverages, and red/processed meat. Scoring for healthy foods/habits is given from 1 to 5 and for unhealthy choices vice versa, with a total score ranging from 8 to 40. A highest score indicates that the individual is closely following the DASH diet guidelines whereas a lower score indicates minimal adherence to the diet [37].

#### 2.6.3. Dietary Inflammatory Index (DII)

The diet’s inflammatory profile was estimated with the use of the DII, computed from the median of the 24 h diet repeated recalls. DII was calculated according to the median reported intake of 29 nutrients of each patient DII nutrients list includes: energy, protein, carbohydrate, total fat, cholesterol, trans fats, mono-unsaturated fatty acids (MUFA), *n*-3 and *n*-6 fatty acids, poly-unsaturated fatty acids (PUFA), alcohol, vitamin B12, vitamin B6, β-carotene, caffeine, fiber, folic acid, niacin, riboflavin, saturated fatty acids (SFA), thiamin, vitamins (A, C, D and E), iron, zinc, selenium, and magnesium. The median dietary consumption of each nutrient was adjusted against reference global intake and then divided by the standard deviation (SD). To calculate DII for each participant, the specific contribution of each nutrient to the DII was calculated by each parameter’s centered proportion, multiplied by the respective parameter’s specific inflammatory effect score. The total DII score for each patient derives by the sum of the 29 nutrients sub scores [38].

### 2.7. Anthropometry

Anthropometric measurements were taken with patients wearing light clothing, according to ISAK recommendations [39]. Body weight (BW) was measured with a digital scale (SECA 813, SECA Group, Hamburg, Germany) with a 0.01 kg precision. Height was measured using a wall-mounted stadiometer (SECA 216, SECA Group, Hamburg, Germany), with a 1 mm precision. Body Mass Index (BMI) was calculated for each participant by dividing body weight in kg with the square of height in meters. Waist circumference (WC) and hip circumference (HC) were also measured and waist to hip circumference ratio was calculated.

### 2.8. Assessment of PA Levels

Self-reported PA was recorded using the International Physical Activity Questionnaire (IPAQ) [40]. The PA of each category and intensity was expressed in metabolic equivalents (METs) and summed for the estimation of the weekly PA. MET intensity tiers were classified IPAQ questions for either vigorous (8 METs) or moderate PA (4 METs) and walking (3.3 METs) [40]. Objectively reported levels of PA were recorded using pedometers (Omron Jog Style HJA 300-EK, Omron Co., Kyoto, Japan) for a 7-day period (same as the self-reported PA). Data from daily average step counts (from the total 7 days period) were converted to PA categories into low (<7500 steps/day), moderate (7500–10,000 steps/daily), or high (>10,000 steps/24 h) PA level [41]. Results from both methods were converted to PA categories for comparable statistical analysis.

### 2.9. Statistical Analyses

Initially, a homogeneity check between the two groups was conducted for the key study variables using the independent samples t-test. The normality of the differences between baseline and post-intervention measurements was then assessed using the Kolmogorov–Smirnov and Shapiro–Wilk tests, as well as through Q–Q plots. To evaluate the effect of the intervention over the 6-month period, a paired samples *t*-test was performed. Effect Size (Cohen’s d for normally distributed variables): 0.2 = small, 0.5 = moderate, 0.8 = large. Additionally, differences in categorical variables were assessed using the chi-square test and Fisher’s exact test. Differences in categorical variables between baseline and 6 months were evaluated using non-parametric methods, specifically the McNemar test and the Marginal Homogeneity test. For consistency in presentation and to avoid misinterpretation, the results are reported as either mean ± standard deviation (SD) or as absolute frequencies (n) accompanied by the corresponding percentages (%). Also, the correlation between the two different methods of assessing PA was examined using Pearson’s correlation coefficient, while Spearman’s rank correlation was applied for ordinal-scale variables. Linear regression analysis was conducted to investigate the extent to which dietary indices were associated with changes in lipid profile outcomes. All statistical tests were two-tailed, with a significance level set at 5% (*p* < 0.05). Statistical analyses were conducted using the Statistical Package for the Social Sciences (SPSS), version 27.0 (IBM, Armonk, NY, USA). For comparisons where the effect size (Cohen’s d) exceeded 0.45 between baseline and 6 months in the intervention group, and 0.5 in the control group, the post hoc power (1 − β) was calculated to be >0.8.

## 3. Results

### 3.1. Baseline Group Comparison

At baseline, no statistically significant differences were found between the two groups regarding the nutritional indexes scores, the lipidemic profiles or the BP levels (*p* > 0.05), as shown in Table 1. However, the ILT group compared to UC group had significantly higher office SBP (142.3 vs. 138.5) mmHg, *p* = 0.013, d = 0.66, and daytime SBP (139.8 vs. 135.1) mmHg, *p* = 0.022, d = 0.61, respectively. Additionally, the ILT group had significantly increased HC compared to the UC group (105.3 vs. 100.6), *p* = 0.029, d = 0.58, and WC/HC ratio (0.9 vs. 1.0), *p* = 0.035, d = 0.56, respectively.

### 3.2. Blood Lipids Profile

**Figure 1:**
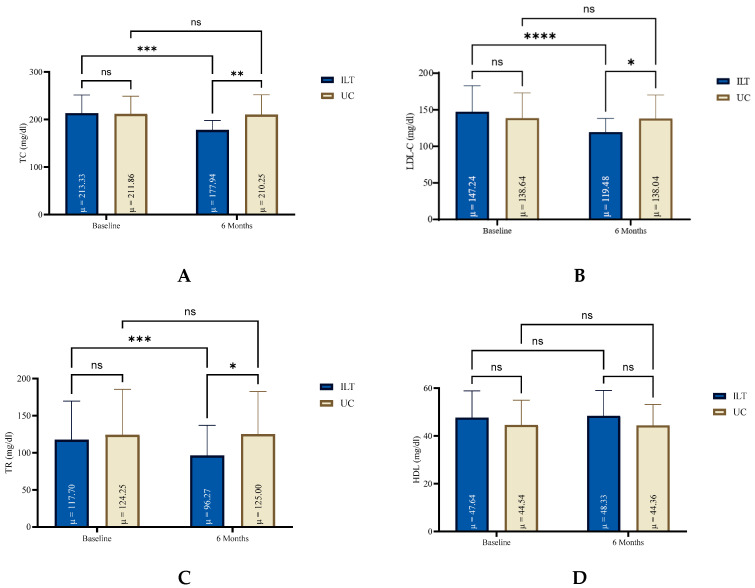
Blood lipid levels for both groups at baseline and at the end of six months lifestyle treatment. HDL: High-density Lipoprotein (**D**); ILT: Intensive Lifestyle Treatment; LDL-C: Low Density Lipoprotein Cholesterol (**B**); ns: non-significant; TC: Total Cholesterol (**A**); TG: Triglycerides (**C**); UC: Usual Care; μ: Mean; * Statistically different compared to baseline measurements (**** *p* < 0.0001, *** *p* = 0.0001 to 0.001, ** *p* = 0.001 to 0.01, * *p* = 0.01 to 0.05, ns: *p* = ≥0.05).

**Figure 2:**
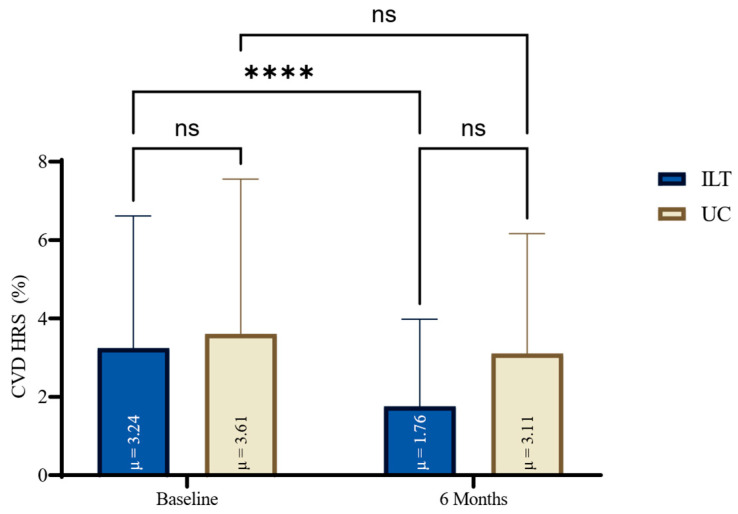
HRS for CVD changes for both groups at baseline and at the end of six months lifestyle treatment. CVD: Cardiovascular Disease; HRS: Hellenic Risk Score; ILT: Intensive Lifestyle Treatment; ns: non-significant; UC: Usual Care; μ: Mean; * Statistically different compared to baseline measurements (**** *p* < 0.0001, ns: *p* = ≥0.05).

At the end of treatment, all lipidemic parameters (TC, TG, LDL-C) except for HDL were significantly reduced in the ILT group (e.g., TC: −35.4 mg/dL, 95%CI: −44.8 to 26.0, *p* < 0.0001, d = 1.33), compared to not significant changes in the UC group, as shown in Figure 1. Also, the ILT group demonstrated significant reduction in CVD risk (−1.5%, 95%CI: −2.1 to −1.0, *p* < 0.0001, d = 1.0) (Figure 2). Results from serum blood lipids alterations can be seen in Table 2, for both groups.

### 3.3. PA Level and Anthropometry Changes

At the end of the study period, BMI (−2.3, 95%CI: −3.0 to −1.5, d = 1.10), WC (−8.4, 95%CI: −10.5 to −6.3, d = 1.43), HC (−8.3, 95%CI: −10.0 to −6.5, d = 1.70), *p* < 0.001 for all, respectively, significantly improved in the ILT group, whereas no significant changes were observed in the UC group for BMI (0.1, 95%CI: −0.4 to 0.6, *p* = 0.691, d = 0.08), for WC (−0.6, 95%CI: −2.8 to 1.6, *p* = 0.580, d = 0.11), or for HC (−0.6, 95%CI: −2.2 to 1.0, *p* = 0.429, d = 0.15). PA levels, objectively recorded by pedometer and expressed as mean steps per day, remained unchanged for the UC group (175.1, 95%CI: −583.5 to 933.6, *p* = 0.640, d = 0.90) whereas for the ILT group, a significant increase was recorded (1096.7, 95%CI: 477.7 to 1715.7, *p* = 0.001, d = 0.69). Results are presented in Table 2.

### 3.4. Dietary Indexes Alterations

**Figure 3:**
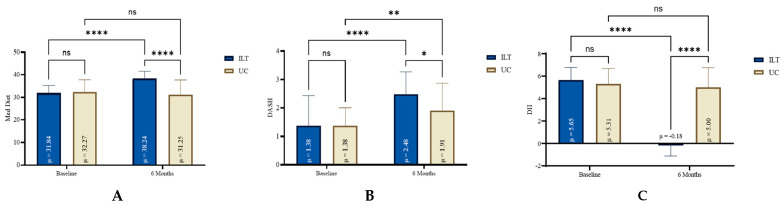
Scoring variation in nutritional indexes for both groups from baseline to 6 months lifestyle treatment; DASH: Dietary Approaches to Stop Hypertension Index (**B**); DII: Dietary Inflammatory Index (**C**); ILT: Intensive Lifestyle Treatment; MedDiet: Mediterranean Diet Index (**A**); ns: non-significant; UC: Usual Care; * Statistically different compared to baseline measurements (**** *p* < 0.0001, ** *p* = 0.001 to 0.01, * *p* = 0.01 to 0.05, ns: *p* = ≥0.05).

During the six months intervention study, dietary adherence improved in the intensified lifestyle modification group, as shown in Table 3. The DASH index significantly improved in the ILT group (1.1, 95%CI: 0.7 to 1.6, *p* < 0.0001, d = 0.89) compared to a more moderate, but significant, improvement in the UC group (0.5, 95%CI: 0.2 to 0.9, *p* < 0.05, d = 0.58). The MedDiet score significantly increased in the ILT group (6.4, 95%CI: 5.5 to 7.3, *p* < 0.0001, d = 2.55), compared to no improvement in the UC group (−1.0, 95%CI: −2.4 to 0.3, *p* = 0.131, d = 0.30). An important and significant improvement was also found in DII score in the ILT group (−5.8, 95%CI: −6.2 to −5.5, *p* < 0.0001, d = 6.10) but not significant changes in the UC group (−0.3, 95%CI; −0.9 to 0.3, *p* = 0.312, d = 0.20). The relative alterations in dietary indexes and scores, for both groups at baseline and at the end of the treatment period, are graphically presented in Figure 3.

### 3.5. Regression Analysis

After assessing single regression analysis, to account for potential confounding factors, multiple linear regression analyses were performed including BMI and mean daily step count as covariates (Table 4). The inclusion of these variables aimed to adjust for differences in BMI and PA, which are known to influence cardiometabolic outcomes. The results remained consistent after adjustment, with key associations, particularly those involving the MedDiet and lipid markers (TC, LDL-C, and TG), remaining statistically significant in the ILT group. Notably, the association between LDL-C and dietary patterns showed some variation compared to earlier models that included only mean daily steps as a covariate, indicating the additional influence of BMI in modulating lipid responses.

Regression analysis revealed a significant and positive association between the DII and CVD HRS in the ILT group (*B* = 0.973, *p* < 0.001), indicating that higher dietary inflammation is associated with increased cardiovascular risk. In contrast, no significant association was observed in the UC group (*B* = 0.050, *p* = 0.819).

Greater adherence to the MedDiet was significantly associated with reductions in TC in both groups, with a stronger effect observed in the ILT group (*B* = −7.238, *p* < 0.001) compared to the UC group (*B* = −3.581, *p* = 0.048). Similarly, LDL-C levels were inversely associated with the MedDiet in both ILT (*B* = −6.431, *p* = 0.004) and UC groups (*B* = −3.974, *p* = 0.014), suggesting a consistent beneficial effect across interventions.

For TG, the ILT group showed a significant negative association with the MedDiet (*B* = −4.103, *p* = 0.035), while no significant effect was observed in the UC group (*B* = 1.033, *p* = 0.695). Interestingly, TG levels were also positively associated with the DASH index in the ILT group (*B* = 9.913, *p* = 0.010), whereas this association was not significant in the UC group (*B* = −6.92, *p* = 0.457). This finding may reflect differential adherence levels or interactions with other dietary factors.

## 4. Discussion

### 4.1. Overall Study Results

This post hoc analysis confirms that ILT in stage 1 HTN patients improves the lipidemic profile and reduces CVD risk. Different nutritional patterns were analyzed based on repeated 24 h dietary recalls in both groups to assess their impact on lipid outcomes. Better adherence to the MedDiet score, DASH index, and DII were significantly associated with beneficial outcomes, but predominantly in the ILT group.

Multiple regression models included BMI and mean daily step count as covariates to control for potential confounding. The inclusion of these variables did not attentively attain the significant associations observed in the ILT group, suggesting that the dietary patterns themselves played a central role in lipid profile improvements. However, LDL-C outcomes did show some variation compared to previous models that included only step count, indicating a possible mediating role of BMI. This emphasizes the value of adjusting for both PA and BMI in dietary studies.

The significant reductions in BMI, WC, and HC in the ILT group underscore the efficacy of an intensive lifestyle intervention in improving anthropometric indices in adults with stage 1 HTN. The absence of significant anthropometric changes in the UC group suggests that UC, following standard lifestyle modification advice, may be insufficient to induce measurable physical improvements over the study period. This emphasizes the need for more structured and supervised interventions to achieve effective BW and WC management in patients with HTN.

Interestingly, the ILT group exhibited a significant decrease in mean daily steps, despite improvements in anthropometry. Along with the not significant alterations in PA levels for the UC group, this may reflect a very low adherence to PA counseling.

The non-significant differences in HDL levels across groups may be attributed to biological factors, such as the slower or more variable response of HDL to dietary interventions. Additionally, methodological limitations, including the short duration of follow-up or potential measurement variability in dietary intake and lipid levels. Another explanation might be a lower adherence to PA recommendations, as HDL levels are affected both by diet and exercise, but exercise seems to have a more pronounced impact on increasing HDL [42,43].

While our findings highlight significant improvements in the lipidemic profile following adherence to specific dietary patterns, it is important to note that short-term changes in lipid levels may not fully reflect their long-term impact on cardiovascular outcomes. Maintenance of normal blood lipid levels over time is known to play a critical role in reducing CVD risk. Although our study demonstrated significant in TC, LDL-C, and TG, the dataset did not allow us to track whether the changes will remain beyond the study period. The duration of normal blood lipid control is particularly important in individuals with HTN, where cumulative exposure to dyslipidemia can induce endothelial dysfunction and atherosclerosis. Therefore, while our results are promising, future longitudinal studies are needed to assess whether these dietary interventions lead to durable lipid improvements and whether this results in reduced clinical events over time.

### 4.2. Mediterranean Diet

The observed positive impact of the adherence to the MedDiet on achieving lower BP levels and improved cardiometabolic parameters aligns with findings for previous studies [44,45,46]. Moreover, as shown in our analysis and corroborated by other research, MedDiet pattern lowers TC and LDL-C levels [47,48,49,50]. In particular, the MedDiet benefits from the high content of nutraceuticals, bioactive compounds, antioxidants, and essential nutrients such as dietary fiber, omega-3 FA, PUFAs, complex carbohydrates, MUFAs, whose synergistic effects contributed to improved management of cardiovascular metabolic risk factors [51,52]. Additionally, higher MedDiet adherence was associated with significant improvement in various lipidemic parameters [30,53,54,55,56]. Adherence to the MedDiet was consistently linked to reductions in TC and LDL-C levels, with stronger associations observed in the ILT group, suggesting enhanced responsiveness to dietary quality under structured intervention.

MedDiet may reduce TC and LDL-C through several mechanisms. High content in polyunsaturated fats from nuts and monounsaturated fats from olive oil enhances lipid metabolism and reduce LDL-C levels. Soluble fiber, found in legumes, fruits, and whole grains binds bile acids, enhancing cholesterol excretion. Plant sterols sourcing from nuts and polyphenols from olive oil and other plant foods may inhibit cholesterol absorption and prevent LDL-C oxidation. Additionally, the low content of saturated fats and refined carbohydrates supports lower hepatic cholesterol production and better blood lipid control [57].

### 4.3. DASH Diet

Another dietary pattern extensively studied for its positive effect on BP and the lipidemic profile of patient with HTN is the DASH diet [58,59]. The DASH diet focuses on increased consumption of food sources rich in potassium, calcium, and magnesium, that seem to improve BP and blood lipid levels [60]. Similarly to our results, it has been previously shown that the DASH dietary pattern has a positive impact on elevated BP, lipidemic profile and CVD risk [61]. Notably, TG levels were influenced by both the Mediterranean and DASH diets in the ILT group, indicating potential interactions between specific dietary patterns and lipid metabolism, even though other studies have shown conflicting results [58].

### 4.4. DII

A wealth of data suggests an association between DII, lipidemic profile, and CVD risk [62,63]. More specifically, the anti-inflammatory effect of this dietary pattern has been shown to exert a positive impact on several cardiometabolic factors, including BP and blood lipids [64]. In this context, a recent study including 10,030 participants showed that the DII may have a potential preventive effect on the 10-year CVD risk [65]. In our study, we confirm a large effect of the DII on the CVD risk of the patients. Higher dietary inflammatory load, as measured by the DII, was positively associated with CVD risk only in the ILT group, underscoring the pro-inflammatory role of diet in CVD pathogenesis when lifestyle factors are actively modified. Based on the above, the anti-inflammatory pattern of diet, expressed by the DII, could be used specifically as a potential clinical tool to reduce the risk of CVD in patients with stage 1 HTN. A possible explanation for the greater improvement in the DII during the study period for the ILT group, compared to the MedDiet score and DASH index, could be the inclusion of higher intake of anti-inflammatory components or exclusion of pro-inflammatory foods, some of which are included in the MedDiet. Also, a better adherence to the regular counseling and the given personalized plans could impact on that remarkable and significant change in the DII. Additionally, misreporting could inflate the observed benefits. In summary, the observed superior improvement of the DII, for the ILT group, could possibly reflect a combination of more targeted anti-inflammatory strategies and greater adherence or personalization, as compared to the broader cardiovascular focus of the Mediterranean and DASH diets.

### 4.5. Intensive Nutritional Treatment

The ILT exhibited a strong impact across several key outcomes, particularly when examined through multiple regression analysis. In the ILT group, significant associations were observed between improved lipid markers (TC, LDL-C, TG) and adherence to dietary patterns, even after adjusting for BMI and PA. These regression results suggest that the intervention strengthened the relationship between diet quality and cardiometabolic outcomes. In contrast, the UC group showed weak or non-significant associations, which may reflect lower adherence or the lack of personalized support.

The regression analyses reinforce the hypothesis that the ILT facilitated not just dietary adherence but also meaningful biological changes. Importantly, the observed effects remained robust in multivariate models, indicating that dietary quality had independent associations with lipid outcomes and CVD risk beyond anthropometric and activity-related influences. These findings align with the concept of precision nutrition, where individualized intervention strategies can amplify the impact of dietary changes on clinical outcomes.

The above findings suggest, as demonstrated elsewhere [17,30], that ILT has a substantial impact on the relationship between dietary patterns and cardiometabolic markers, enhancing the protective effects of the Mediterranean and DASH diets on blood lipids and revealing the detrimental association of a high inflammatory dietary profile with CVD risk. In contrast, in the UC group the relationships were weak or non-significant, possibly due to the absence of structured support and lower dietary compliance.

These results support the importance of holistic lifestyle modification and suggest that dietary quality, when combined with PA and weight management, exerts a synergistic effect on CVD risk reduction.

### 4.6. Limitations and Strengths

This study has several limitations that may impact on the generalizability of our findings. First, the sample size in each group minimizes the statistical power. Second, the large number of variables hindered the statistical analysis due to the sample number of the groups. Third, our study participants were predominantly white Caucasians from the Greek area which may not be applicable to other populations. Moreover, reliance on self-reported data for many variables and covariates, including nutritional information, increases the possibility of recall bias. Blinding of outcome assessors was not ensured in this study, which may have introduced some degree of measurement bias, particularly in the assessment of subjective or modifiable outcomes.

All-important covariates tested did not differ between the post hoc analysis groups at baseline. However, the ILT group started with significantly higher day systolic ABPM levels at baseline compared to the UC control group. On the contrary, exclusion of the patients prescribed with antihyperlipidemic medication increased the reliability of the results and the present post hoc analysis.

We acknowledge that the sample size of the current post hoc analysis is relatively small (n = 33 in the ILT group and n = 28 in the UC group), which may indeed increase the risk of both type I and type II errors, particularly in subgroup and regression analyses. To address this concern, we performed an effect size calculation for each primary comparison. For comparisons where the effect size (Cohen’s d) exceeded 0.45 between baseline and 6 months in the intervention group, and 0.5 in the control group, the post hoc power (1 − β) was calculated to be >0.8. This suggests that the study had adequate power to detect medium effect sizes in these comparisons.

Another important consideration is the variability in patient response to dietary interventions based on the presence of comorbid conditions. Individuals with metabolic disorders or inflammatory conditions may have altered lipid metabolism, insulin sensitivity, or inflammatory pathways that can influence both efficacy and mechanisms of nutritional interventions. In our study, dietary patterns were analyzed without stratifying participants by comorbid status, which may limit the generalizability of the findings to more complex patient populations. Given the increasing interest in personalized nutrition, future research should consider stratified or subgroup analyses to highlight strategies for customized recommendations, especially in clinical practice where comorbidities often coexist.

## 5. Conclusions

In summary, the findings of this post hoc analysis of the HINTreat RCT indicate that ILT in newly diagnosed patients with stage 1 HTN significantly improves lipid profiles and reduces CVD risk. Regression analysis revealed that these improvements were closely associated with adherence to specific dietary patterns, namely, the MedDiet (linked to TC and LDL reductions), the DASH diet (associated with lower TG), and low-inflammatory diets (associated with reduced CVD risk).

These associations were statistically significant primarily in the ILT group and remained robust after adjusting for BMI and mean daily step count, highlighting the independent effect of dietary quality. The absence of similar findings in the UC group underscores the need for personalized and structured intervention to achieve clinically meaningful improvements.

Thus, while the choice of dietary pattern plays a crucial role, the intensity and personalization of the intervention appear to be also decisive factors in translating dietary adherence into measurable cardiometabolic benefits. Regression modeling confirms that precision nutrition, incorporating individualized plans, lifestyle monitoring, and clinical support is more effective than general dietary advice for high-risk individuals.

These findings support the integration of intensive, personalized dietary counseling into standard care for patients with early-stage HTN and suggest that diet-focused interventions, when coupled with behavioral support, can independently and significantly influence key cardiovascular risk markers.

Our results hold particular relevance for real-world clinical practice and primary care, where stage 1 HTN is commonly diagnosed and managed. The demonstrated impact of dietary pattern adherence, particularly when supported through intensive, personalized intervention, underscores the value of integrating precision nutrition in cardiovascular management. Primary care practitioners are well-positioned to deliver targeted lifestyle counseling, monitor dietary adherence, and support behavior change. Dietary interventions can play a key role as a practical and effective component of early cardiovascular risk reduction and improve lipidemic profile.

Although large-scale trials are still needed to assess the impact of dietary patterns on CVD risk and lipidemic profile in patients with stage 1 HTN, current observational evidence plays a key role in guiding dietary recommendations to promote specific hyperlipidemia treating-related eating habits.

## Figures and Tables

**Table 1:** Patients characteristics at baseline for both groups.

		Baseline			
		ILT (n = 33)	UC (n = 28)		
		Mean ± SD	Mean ± SD	*p*	Cohens’ d
Anthropometric Indices					
Weight (kg)		86.4 ± 16.7	85.9 ± 14.5	0.891	0.04
Height (cm)		1.7 ± 0.1	1.7 ± 0.1	0.387	0.22
BMI (kg/m^2^)		29.8 ± 5.2	28.9 ± 4.3	0.475	0.19
WC (cm)		97.9 ± 13.6	100.0 ± 12.1	0.540	0.16
HC (cm)		105.3 ± 8.8	100.6 ± 7.3	0.029	0.58
WC/HC		0.9 ± 0.1	1.0 ± 0.1	0.035	0.56
Nutritional Indexes Scores					
DASH		1.4 ± 1.1	1.4 ± 0.6	0.986	0.00
DII		5.7 ± 1.1	5.3 ± 1.4	0.284	0.28
MedDiet		31.8 ± 3.4	32.3 ± 5.6	0.724	0.1
Physical Activity					Effect size
Pedometer PA categories *					
1		0.8%	0.7%	0.266	0.21 ^a^
2		18.2%	0.2%		
3		0.0%	0.1%		
IPAQ PA categories **					
1		51.5%	57.1%	0.054	0.28 ^b^
2		39.4%	17.9%		
3		9.1%	25.0%		
					Cohens’ d
Mean steps per day (pedometer)		6573 ± 231	6168 ± 1929	0.471	0.19
Blood Pressure					
Office BP	SBP (mmHg)	143.2 ± 5.5	138.5 ± 8.6	0.013	0.66
	DBP (mmHg)	90.3 ± 9.1	88.2 ± 7.1	0.316	0.26
ABPM	Daytime SBP (mmHg)	139.8 ± 8.9	135.1 ± 6.3	0.022	0.61
	Daytime DBP (mmHg)	92.5 ± 9.8	88.2 ± 7.4	0.063	0.49
Lipidemic Profile and CVD HRS					
TC (mg/dL)		213.3 ± 38.2	212.0 ± 37.3	0.880	0.04
TG (mg/dL)		117.7 ± 52.1	124.3 ± 61.4	0.653	0.12
HDL (mg/dL)		47.6 ± 11.2	44.5 ± 10.6	0.271	0.29
LDL-C (mg/dL)		147.2 ± 35.6	138.6 ± 34.5	0.345	0.25
					Effect size
CVD HRS (%)		3.2 ± 3.4	3.6 ± 4.0	0.699	0.1
CVD HRS Category ***					
1		3.0%	3.6%	0.542	0.19 ^a^
2		69.7%	71.4%		
3		24.2%	14.3%		
4		3.0%	10.7%		
Dyslipidemia ****					
0		0.0%	10.7%	0.054	0.25 ^b^
1		100.0%	89.3%		

^a^: Cramér’s V (V); ABPM: Ambulatory Blood Pressure Monitoring; ^b^: Phi Coefficient (φ); BMI: Body Mass Index; BP: Blood Pressure; CVD: Cardiovascular Disease; DASH: Dietary Approaches to Stop Hypertension; DBP: Diastolic blood pressure; DII: Dietary Inflammatory Index; HC: Hip Circumference; HDL: High Density Lipoprotein; HRS: Hellenic Risk Score; ILT: Intensive Lifestyle Treatment; IPAQ: International Physical Activity Questionnaire; LDL-C: Low Density Lipoprotein Cholesterol; MedDiet: Mediterranean Diet; PA: Physical Activity; SBP: Systolic blood pressure; TC: Total Cholesterol; TG: Triglycerides; UC: Usual Care; WC: Waist Circumference; * Pedometer PA Categories: 1: low (<7500 steps/day), 2: moderate (7500–10,000 steps/daily), 3: high PA (>10,000 steps/24 h); ** IPAQ PA Categories: 3: vigorous (8 METs), 2: moderate PA (4 METs), 1: low (3.3 METs); *** Hellenic Risk Score Categories: 1: Low Risk (LR) = <1%, 2: Moderate Risk (MR) = ≥1% and <5%, 3: High Risk (HR) = ≥5% and <10%, 4: Very High Risk (VHR) = ≥10%; **** 1: yes, 0: no.

**Table 2:** Within group comparison at baseline and at six months study period.

	ILT (n = 33)				UC (n = 28)			
	Baseline	6 Months			Baseline	6 Months		
Variable	Mean Dif ± SEM	95% CI	*p*	Cohen’s d	Mean Dif ± SEM	95% CI	*p*	Cohen’s d
Anthropometry								
Weight (kg)	−6.6 ± 1.1	[−8.7 to −4.4]	<0.001	1.08	0.4 ± 0.8	[−1.2 to 2.0]	0.618	0.10
BMI (kg/m^2^)	−2.3 ± 0.4	[−3.0 to −1.5]	<0.001	1.10	0.1 ± 0.3	[−0.4 to 0.6]	0.691	0.08
WC (cm)	−8.4 ± 1.0	[−10.5 to −6.3]	<0.001	1.43	−0.6 ± 1.1	[−2.8 to 1.6]	0.580	0.11
HC (cm)	−8.3 ± 0.9	[−10.0 to −6.5]	<0.001	1.70	−0.6 ± 0.8	[−2.2 to 1.0]	0.429	0.15
Lipidemic Profile								
TC (mg/dL)	−35.4 ± 4.6	[−44.8 to −26.0]	<0.0001	1.33	−1.6 ± 6.4	[−14.8 to 11.6]	0.804	0.05
TG (mg/dL)	−21.4 ± 5.0	[−31.5 to −11.3]	<0.0001	0.75	0.8 ± 8.1	[−15.8 to 17.3]	0.927	0.02
HDL (mg/dL)	0.7 ± 1.0	[−1.2 to 2.6]	0.470	0.13	−0.2 ± 1.0	[−2.2 to 1.8]	0.855	0.04
LDL-C (mg/dL)	−27.8 ± 5.7	[−39.0 to −16.1]	<0.0001	0.85	−0.6 ± 5.7	[−12.2 to 11.0]	0.915	0.02
CVD HRS (%)	−1.5 ± 0.3	[−2.0 to −1.0]	<0.0001	1.00	−0.5 ± 0.3	[−1.1 to 0.1]	0.114	0.31
CVD HRS Category * (n)				Effect size				Effect size
1	8		<0.0001	4	0		0.180	1.34
2	0				1			
3	−8				1			
4	0				−2			
Dyslipidemia ** (n)								
0	10		0.002	3.0	0		1.000	0.5
1	−10				0			
Physical Activity								
Pedometer PA categories ***	(n)							
1	−4		0.046	1.6	0		0.109	0.38
2	2				1			
3	2				−1			
IPAQ PA categories ****	(n)						0.705	0.24
1	−5		0.808	2.0	−1			
2	2				3			
3	3				−2			
Mean steps per day (pedometer)	1096.7 ± 303.9	[477.7 to 1715.7]	0.001	0.69	175.1 ± 369.7	[−583.5 to 933.6]	0.640	0.90

BMI Body Mass Index; CVD: Cardiovascular Disease; HC: Hip Circumference; HDL: High Density Lipoprotein; HRS: Hellenic Risk Score; ILT: Intensive Lifestyle Treatment; IPAQ: International Physical Activity Questionnaire; LDL-C: Low Density Lipoprotein Cholesterol; PA: Physical Activity; TC: Total Cholesterol; TG: Triglycerides; UC: Usual Care; WC: Waist Circumference; * Hellenic Risk Score Categories: 1: Low Risk (LR) = <1%, 2: Moderate Risk (MR) = ≥1% and <5%, 3: High Risk (HR) = ≥5% and <10%, 4: Very High Risk (VHR) = ≥10%; ** 1: yes, 0: no; *** Pedometer PA Categories: 1: low (<7500 steps/day), 2: moderate (7500–10,000 steps/daily), 3: high PA (>10,000 steps/24 h); **** IPAQ PA Categories: 3: vigorous (8 METs), 2: moderate PA (4 METs), 1: low (3.3 METs).

**Table 3:** Within group comparison for the nutritional indexes at baseline and at six months study period.

	ILT (n = 33)				UC (n = 28)			
	Baseline	6 Months			Baseline	6 Months		
Variable	Mean Dif ± SEM	95% CI	*p*	Cohen’s d	Mean Dif ± SEM	95% CI	*p*	Cohen’s d
DASH	1.1 ± 0.2	[0.7 to 1.6]	<0.0001	0.89	0.5 ± 0.2	[0.2 to 0.9]	0.005	0.58
DII	−5.8 ± 0.2	[−6.2 to −5.5]	<0.0001	6.10	−0.3 ± 0.3	[−0.9 to 0.3]	0.312	0.20
MedDiet	6.4 ± 0.4	[5.5 to 7.3]	<0.0001	2.55	−1.0 ± 0.7	[−2.4 to 0.3]	0.131	0.30

DASH: Dietary Approaches to Stop Hypertension; DII: Dietary Inflammatory Index; ILT: Intensive Lifestyle Treatment; MedDiet: Mediterranean Diet; UC: Usual Care.

**Table 4:** Regression analysis for the effect of different dietary patterns on the lipidemic profile and CVD risk, for both groups.

			Unstandardized Coefficients		Standardized Coefficients			
			B	Std. Error	Beta		*p*	95% C.I. for B
ILT	CVD HRS	DII	0.973	0.203	0.628	4.786	<0.001	[0.6 to 1.4]
	TC	Med Diet	−7.238	1.383	−0.684	−5.235	<0.001	[−10.1 to −4.4]
	TG	Med Diet	−4.103	1.85	−0.362	−2.218	0.035	[−7.9 to −0.3]
	TG	DASH	9.913	3.618	0.433	2.74	0.010	[2.5 to 17.3]
	LDL-C	Med Diet	−6.431	2.063	−0.492	−3.117	0.004	[−10.7 to −2.2]
UC	CVD HRS	DII	0.05	0.215	0.048	0.232	0.819	[−0.4 to 0.5]
	TC	Med Diet	−3.581	1.722	−0.365	−2.08	0.048	[−7.1 to 0.0]
	TG	Med Diet	1.033	2.603	0.084	0.397	0.695	[−4.4 to 6.4]
	TG	DASH	−6.92	9.157	−0.151	−0.756	0.457	[−25.8 to 12.0]
	LDL-C	Med Diet	−3.974	1.505	−0.46	−2.641	0.014	[−7.1 to −0.9]

CVD: Cardiovascular Disease; DASH: Dietary Approaches to Stop Hypertension; DII: Dietary Inflammatory Index; HRS: Hellenic Risk Score; ILT: Intensive Lifestyle Treatment; LDL-C: Low Density Lipoprotein Cholesterol; MedDiet: Mediterranean Diet; TC: Total Cholesterol; TG: Triglycerides; UC: Usual Care. Multiple regression models included both BMI and mean daily step count as covariates.

## Data Availability

All data are available upon request to the first author.

## Abbreviations

The following abbreviations are used in this manuscript:

ABPM	Ambulatory Blood Pressure Monitoring
ASCVD	Atherosclerotic Cardiovascular Disease
BMI	Body Mass Index
BP	Blood Pressure
BW	Body Weight
CVD	Cardiovascular Disease
DASH	Dietary Approaches to Stop Hypertension
DBP	Diastolic Blood Pressure
DII	Dietary Inflammatory Index
DM	Diabetes Mellitus
EH	Essential Hypertension
ESH	European Society of Hypertension
HC	Hip Circumference
HDL	High Density Lipoprotein
HRS	Hellenic Risk Score
HTN	Hypertension
ILT	Intensive Lifestyle Treatment
LDL	Low Density Lipoprotein
MedDiet	Mediterranean Diet
MUFA	Monounsaturated Fatty Acids
Na	Sodium
PA	Physical Activity
PUFA	Polyunsaturated Fatty Acids
RCT	Randomized Controlled Trial
SBP	Systolic Blood Pressure
SFA	Saturated Fatty Acids
TC	Total Cholesterol
TG	Triglycerides
UC	Usual Care
WC	Waist Circumference
